# Wrist-ankle acupuncture for primary dysmenorrhea: a randomized controlled trial evaluating the efficacy of an analgesic strap

**DOI:** 10.3389/fneur.2024.1362586

**Published:** 2024-05-30

**Authors:** Shujie Zhai, Chenmiao Wang, Yi Ruan, Yue Liu, Rui Ma, Fanfu Fang, Qinghui Zhou

**Affiliations:** ^1^Department of Rehabilitation Medicine, Eastern Hepatobiliary Surgery Hospital, Naval Medical University, Shanghai, China; ^2^School of Traditional Chinese Medicine, Naval Medical University, Shanghai, China; ^3^Department of Acupuncture and Moxibustion, Changhai Hospital, Naval Medical University, Shanghai, China; ^4^Department of Cardiology, Eastern Hepatobiliary Surgery Hospital, Naval Medical University, Shanghai, China; ^5^Department of Rehabilitation Medicine, Changhai Hospital, Naval Medical University, Shanghai, China

**Keywords:** primary dysmenorrhea (PD), wrist-ankle acupuncture (WAA), analgesic, acupressure wrist-ankle strap, RCT (randomised controlled trial)

## Abstract

**Background:**

Drawing on the principles of wrist-ankle acupuncture (WAA), our research team has developed a portable device for WAA point compression, termed the acupressure wrist-ankle strap (AWA). The current study aims to evaluate the efficacy of the AWA in alleviating pain associated with primary dysmenorrhea.

**Methods:**

A single-blind, randomized clinical trial was conducted from April 1, 2019, to December 31, 2019. 78 participants with primary dysmenorrhea were recruited from Shanghai University of Traditional Chinese Medicine. All participants were treated on the first day of menstruation for 30 min. Participants in the AWA group used the AWA, the internal side of which is equipped with a tip compression component, while participants in the non-acupressure wrist-ankle acupuncture(NAWA)group used the NAWA, with the inside tip pressing parts removed. The main outcome was the difference in visual analogue scale (VAS) score between baseline and 30 minutes after randomization.

**Results:**

A total of 78 participants aged 18 to 30 years were included in the intention-to-treat analyses. The VAS scores (mean [standard deviation]) in the AWA group were significantly lower than those in the NAWA group at each time point of intervention (5 minutes: 95% CI, [−1.27 to −0.68], *p* < 0.001; 10 minutes: 95% CI, [−2.34 to −1.51], *p* < 0.001; 30 minutes: 95% CI, [−3.74 to −2.72], *p* < 0.001). In the AWA group, 16 participants reported “obvious relief” of dysmenorrhea pain while 23 did not; the average onset time of analgesia they reported were (21.50 ± 3.65) min, while no subjects in NAWA group reported obvious pain relief. The pain threshold (mean [standard deviation]) at SP9 of both sides in AWA group decreased significantly after intervention that in NAWA group (Left: 95% CI, [−5.02 to −1.81], *p* < 0.001; Right: 95% CI, [−7.67 to −4.24], *p* < 0.001). There was no significant change in the temperature at CV4 in either group (95% CI, [−0.63 to −0.66], *p* = 0.970).

**Conclusion:**

This trial substantiates our hypothesis that the AWA provides immediate analgesic effects. The AWA represents an effective and safe non-invasive physical therapy option, which patients can self-administer to alleviate abdominal pain

## 1 Introduction

Dysmenorrhea is one of the most prevalent causes of pelvic pain among women (1). The World Health Organization categorized it as a primary and non-specific condition in the Tenth Revision of the International Statistical Classification of Diseases and Related Health Problems in 1992 (2). Dysmenorrhea manifests in two forms: primary and secondary, with primary dysmenorrhea being the more common. It is characterized by lower abdominal pain occurring just before or during menstruation, without any evident pelvic pathology, typically emerging 6–12 months or 1–2 years following menarche (3, 4). Secondary dysmenorrhea accounts for about 10% of dysmenorrhea cases, and the most common cause is endometriosis (5). The pain associated with dysmenorrhea can range from moderate to severe and significantly impairs quality of life, affecting approximately 72.8% of women (2).

The etiology of primary dysmenorrhea primarily involves prostaglandins (6). Currently, the most widely used pharmacological treatments for dysmenorrhea are non-steroidal anti-inflammatory drugs (NSAIDs) (7), which inhibit prostaglandin synthesis via cyclooxygenase inhibition (8). Another common treatment is oral contraceptives, which suppress ovulation and reduce endometrial thickness, thereby alleviating menstrual cramps and pain (9, 10). However, pharmacological treatments can lead to gastrointestinal side effects, impact the central nervous system, affect metabolism, have diminished long-term effectiveness, and may lead to drug resistance (11). Alternative treatments for menstrual cramps include: (a) transcutaneous electrical nerve stimulation, which modifies the body’s pain perception (12), (b) acupuncture, which increases the release of norepinephrine and β-endorphins into the bloodstream and cerebrospinal fluid, reducing pain (13), and (c) surgical interventions like laparoscopic uterosacral nerve ablation. Each method offers benefits but also carries potential side effects. Currently, about 70% of women with menstrual cramps engage in self-management (14, 15). Factors such as an unhealthy lifestyle, lack of self-care awareness, and chronic repetitive menstrual pain can exacerbate symptoms by inducing menstruation-related fear or anxiety.

Given the severity of symptoms during dysmenorrhea episodes, which often prevents timely medical consultation, and the challenges patients face in mastering effective pain management techniques, there is a significant practical need for an effective, patient-administered physical therapy. Numerous studies have demonstrated the efficacy of acupressure in alleviating dysmenorrhea (16–18). Acupressure is a non-invasive method that involves applying pressure to specific acupoints using fingers or thumbs to relieve pain. Wrist-ankle acupuncture (WAA), a specific modality within the broader acupuncture framework, also shows promising analgesic effects (19–21). Our research team has developed the Acupressure Wrist-Ankle Strap (AWA) based on WAA principles. This innovative, portable device (22) aims to replace traditional acupuncture needles, allowing patients to self-administer treatment and achieve pain relief. However, its effectiveness in treating primary dysmenorrhea had not been previously verified. To support and validate the clinical efficacy of this novel therapy, we designed a single-blind randomized controlled trial (RCT) to assess the effectiveness of the AWA and to explore potential factors influencing its efficacy.

## 2 Methods

### 2.1 Study design and protocol

This is a single-blind RCT investigating the immediate pain-relieving effect and safety of AWA in young women with primary dysmenorrhea. Written informed consent was obtained from all participants. Participants were randomly assigned into 2 groups, in a 1:1 ratio, receiving AWA (intervention group) and non-acupressure wrist-ankle strap (NAWA, sham group), respectively. The study protocol was approved by the Chinese Ethics Committee of Registering Clinical Trials (Ethics Reference: ChiECRCT20190037) and registered in the Chinese Clinical Trial Registry (ID: ChiCTR1900021727) on March 7, 2019, and implemented in accordance with the Declaration of Helsinki. Study design and reporting were in accordance with the recommendations of the CONSORT Statement (23) and the Standards for Reporting Interventions in Clinical Trials of Acupuncture (STRICTA) (24). The protocol was published previously (25).

### 2.2 Study participants

Study participants were recruited from Shanghai University of Traditional Chinese Medicine (Shanghai, China). Participants were enrolled if they fulfilled the following criteria: women aged 18 to 30 years; meeting the criteria for the diagnosis of primary dysmenorrhea based on the Primary Dysmenorrhea Consensus Guidelines (26); basic regularity of the menstrual cycle ([28 ± 7] days); no ongoing treatment for dysmenorrhea; mean pain intensity score evaluated by the visual analogue scale (VAS) of >2 for 3 consecutive menstrual cycles. Patients with any of the following conditions were excluded: pregnancy, prepregnancy, or lactation; having experienced miscarriage or stillbirth; having cardiovascular, hepatic, renal, hematopoietic, and other serious primary diseases or mental disorders; being suspected of having other diseases based on the medical history; having taken any analgesic medications within 24 h before the intervention.

### 2.3 Interventions

**Figure 1:**
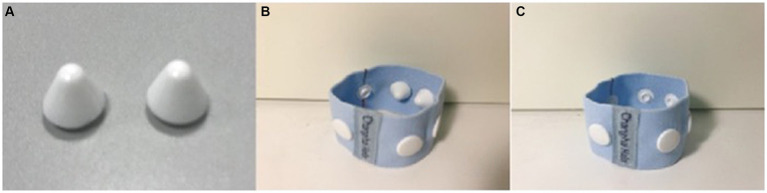
Compression part **(A)**, wrist-ankle strap with compression part **(B)**, and wrist-ankle strap of removing the compression part **(C)**.

**Figure 2:**
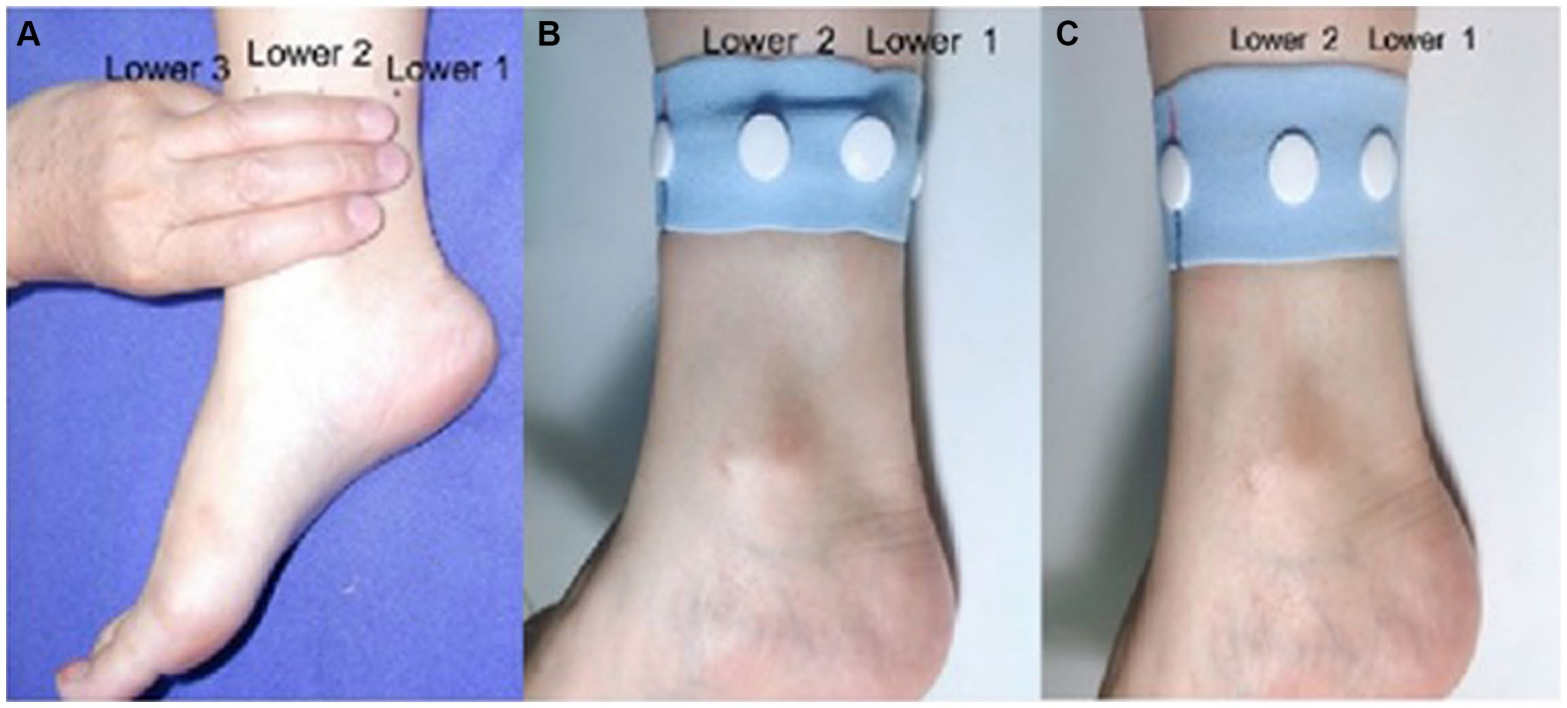
Anatomical location of lower 1 and lower 2 **(A)**. Unilateral schematic diagram of the acupressure wrist-ankle strap **(B)** and unilateral non-compressed wrist-ankle strap **(C)**.

Interventions were administered as described in the previously published protocol (25). During the intervention, the participants were in a supine position. The acupuncturist was responsible for setting up the intervention device, and putting it in place for the participants. In AWA group, AWA was used to replace the stimulation of acupuncture needles. Two compression parts were installed inside the wrist-ankle strap (Figure 1), and patients wore a wrist-ankle strap on each ankle for 30 min, so that both the Lower 1 and Lower 2 compression points were simultaneously pressed (Figure 2). For NAWA group, patients wore a wrist-ankle strap on each ankle for 30 min the same as the intervention group, but the compression parts were not installed on the inside of the straps.

If the participants were still in severe pain after 30-min intervention with the wrist-ankle strap, a variety of follow-up treatments would be provided, including traditional acupuncture, moxibustion, or analgesic medications. Participants with severe continuous pain after the above treatment would be referred to a general physician or gynecologist to receive other form of treatment.

### 2.4 Measures

All participants were instructed to complete the general information form, and the Cox Menstrual Symptom Scale (CMSS) (27) at the enrollment. Outcomes were measured as described in the previously published protocol (25).

The primary outcome was the change in pain intensity of primary dysmenorrhea measured by VAS (score range, 0–10; higher scores indicate greater pain) from baseline to after 30-min intervention.

The secondary outcomes were as follows: (1) onset time of analgesia, defined as the time taken for the dysmenorrhea pain to be “obviously relieved” as reported by the participants; (2) pain threshold measurement, measured at the Yinlingquan acupoint (SP9) using a pressure pain detector (YISIDA, Hong Kong, China) at 3 min before intervention and at the end of intervention (after removing the wrist-ankle strap); (3) skin temperature at Guanyuan acupoint (CV4), measured using an infrared thermal imager (FLIR ONE, FLIR System, Wilson, Oregon, United States) at 3 min before intervention, and at the end of intervention (after removing the wrist-ankle strap); (4) patients’ expectation and satisfaction with the outcome, investigated using the Expectation and Treatment Credibility Scale (ETCS) (28). The ETCS includes 4 statements with a Yes/No response. ETCS1: I am sure this treatment will relieve my pain; ETCS2: I think this treatment is reasonable; ETCS3: I will recommend this therapy to my friends; ETCS4: I believe this therapy can cure other diseases. The ETCS questionnaire was completed 3 min before intervention and 3 min after the 30-min intervention.

### 2.5 Sample size calculation

Based on the preliminary experiments, the mean baseline VAS score was 74 mm with a standard deviation of 14 mm. The researchers expected the mean difference in the VAS score between the two groups (D1) would be 10 mm and the correlation between measurement points (ρ) would be 0.7, which was not moderately clinically meaningful but represented a minimally important difference. With the test levels set at α = 0.05 (2-sided), β = 0.1, D1 = 1, M = 4, s = 1.4, r = 0.7, and the module selected as “tests for two means in a repeated measures design,” where α represents the probability of rejecting a true null hypothesis, β is the probability of obtaining a false negative with the statistical test, and M is the number of repeated measurements, finally, at least 32 participants were required per group. Considering a 20% dropout rate, 39 participants would be enrolled in each group, giving a total of 78 participants.

### 2.6 Randomization and blinding

Eligible participants were randomly assigned in an equal ratio to the AWA group and NAWA group via a central randomization system. The participants and the researchers who evaluated the results were unaware of which intervention group the participants had been assigned to. The wrist-ankle straps used in both groups had the same packaging. As only one 30-min intervention was given, the participants were not aware of the differences between the products during the intervention.

### 2.7 Statistical analysis

Baseline characteristics and clinical outcomes are described on the basis of the intention-to-treat population (*N* = 78). The value of missing data was defined based on the last-observation-carried-forward principle. Measurement data were expressed as mean ± standard deviation, while count data were described as number and percentage. The normal distribution test and homogeneity test of variance were used for measurement data. The baseline characteristics of the 2 groups were analyzed using *t* test or nonparametric test. If the baseline characteristics were imbalanced, covariance analysis was used to adjust the baseline characteristics. Repeated measures analysis of variance was adopted to analyze the outcome (analgesic effect) between groups and observed time. All statistical tests were 2-sided, and the difference is considered statistically significant when *p* < 0.05.

## 3 Result

### 3.1 Baseline characteristics of the participants

**Figure 3:**
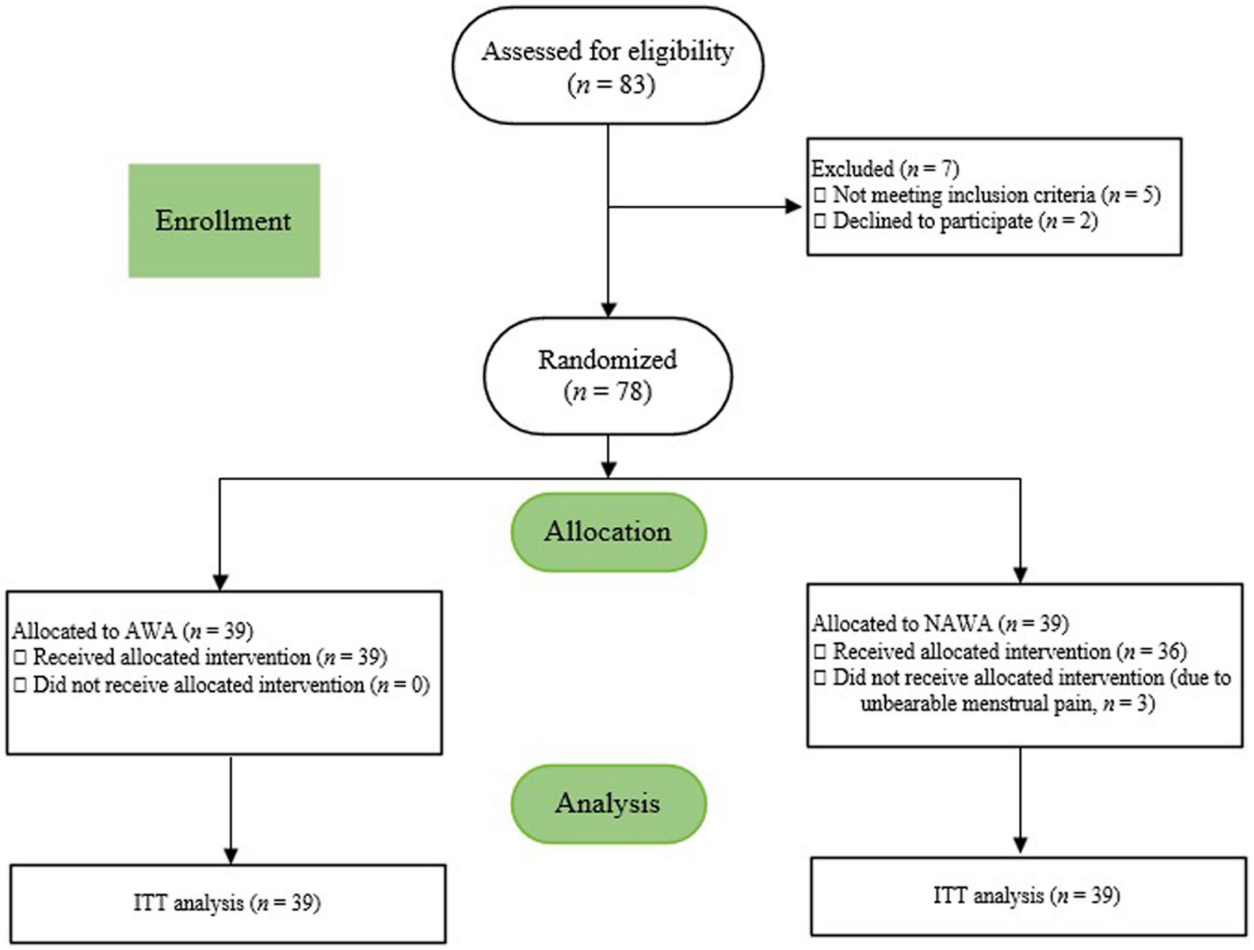
CONSORT flow diagram. AWA, acupressure wrist-ankle strap; NAWA, non-acupressure wrist-ankle strap. ITT, intention-to-treat.

From April 1, 2019 to December 30, 2019, 85 women were screened for eligibility; 78 qualified and underwent randomization. Three participants in NAWA group asked for analgesic medication after assignment due to their unbearable menstrual pain, and so did not accept the wrist-ankle strap intervention. A total of 75 participants provided complete data on the outcome measures; 78 were included in the intention-to-treat analyses (Figure 3). Tables 1, 2 show the characteristics and dysmenorrhea symptoms of the participants at baseline before intervention.

**Table 1:** Demographic and clinical characteristics of the participants at baseline.

Item	AWA group (*n* = 39)	NAWA group (*n* = 39)	*p* value
Age (mean ± SD, year)	23.67 ± 3.41	23.54 ± 2.86	0.857
BMI (mean ± SD, kg/m^2^)	20.86 ± 3.08	20.14 ± 2.04	0.223
Age of menarche (mean ± SD, year)	13.69 ± 1.73	12.95 ± 1.10	0.027
Menstrual cycle (*n*[%])			0.250
≤ 21 days	0	0	
22–35 days	29 (74.4%)	34 (87.2%)	
≥ 36 days	10 (25.6%)	5 (12.8%)	
Irregular	0	0	
Menstrual period (*n*[%])			1.000
1–3 days	2 (5.1%)	2 (5.1%)	
4–7 days	36 (92.3%)	36 (92.3%)	
8–14 days	1 (2.6%)	1 (2.6%)	
≥ 15 days	0	0	
First experience of dysmenorrhea (*n*[%])			0.771
Appears immediately after menarche	8 (20.5%)	10 (25.6%)	
Within 1–2 years after menarche	19 (48.7%)	13 (41.0%)	
In recent 3 years	12 (30.8%)	13 (33.3%)	
Taking medicine	0	3 (7.7%)	0.240

BMI, body mass index; SD, standard deviation; AWA, acupressure wrist-ankle strap; NAWA, non-acupressure wrist-ankle strap.Three participants in the NAWA group took drugs: hydroxychloroquine sulfate to treat Sjogren’s syndrome, metronidazole to treat periodontitis, and compound licorice mixture to treat cough; neither affects the intervention during the trial period.

**Table 2:** Dysmenorrhea symptom scores of CMSS of the participants at baseline (P_50_ [P_25_, P_75_]).

Symptom	AWA group (*n* = 39)	NAWA group (*n* = 39)	*p* value
Pain in the lower abdomen	3 (2,3)	3 (2,3)	0.838
Nausea	1 (0,2)	0 (0,1)	0.067
Vomiting	0 (0,1)	0 (0,1)	0.420
Inappetence	2 (1,2)	1 (1,2)	0.255
Headache	0 (0,1)	0 (0,1)	0.776
Back (lumbosacral) pain	2 (1,3)	2 (1,2)	0.321
Leg pain	0 (0,1.5)	0 (0,1)	0.495
Fatigue	2 (1,3)	1 (1,2.5)	0.488
Vertigo	0 (0,1)	0 (0,2)	0.659
Diarrhea	1 (1,2)	1.5 (0.5,2)	0.885
Complexion change	1 (1,2)	1 (1,2)	0.689
Stomachache	0 (0,1)	0 (0,0.5)	0.322
Face flushing	0 (0,0)	0 (0,0)	0.851
Insomnia	0 (0,1)	0 (0,1.5)	0.957
Body pain	0 (0,1)	0.5 (0,2)	0.791
Depression	0 (0,1)	0.5 (0,1)	0.597
Irritable	1 (0.5,2)	1 (1,2)	0.834
Neuroticism	0 (0,1)	1 (0,1.5)	0.244
Total score	18 (12,23)	16 (13,25)	0.836

CMSS, Cox Menstrual Symptom Scale; AWA, acupressure wrist-ankle strap; NAWA, non-acupressure wrist-ankle strap.

### 3.2 Primary outcome

Between-group comparisons of VAS scores showed that with the extension of the intervention time, the between-group differences were statistically significant at 5, 10 and 30 min (5 min: 95% CI, [−1.27 to −0.68], *p* < 0.001; 10 min: 95% CI, [−2.34 to −1.51], *p* < 0.001; 30 min: 95% CI, [−3.74 to −2.72], *p* < 0.001) (Table 3). The VAS score (mean [standard deviation]) changes in the AWA group decreased significantly at each time point (5 min: 4.72[1.84] vs 5.72 [1.75], *p* < 0.05; 10 min: 3.95[1.93] vs 5.72 [1.75], *p* < 0.05; 30 min: 2.82[2.01] vs 5.72 [1.75], *p* < 0.05), while the VAS score of NAWA group had no significant difference (5 min: 5.05 [1.50] vs 5.08 [1.46], *p* > 0.05; 10 min: 5.23[1.50] vs 5.08 [1.46], *p* > 0.05; 30 min: 5.41 [1.60] vs 5.08 [1.46], *p* > 0.05) (Table 3).

**Table 3:** Outcome measurements during the study.

Variable	AWA group (*n* = 39)	NAWA group (*n* = 39)	*p* value	Difference between groups	
				Effect size (95% CI)	*p* value
Pain severity of primary dysmenorrhea, VAS score (mean ± SD)					
Baseline	5.72 (1.75)	5.08 (1.46)	0.082		
5 min after intervention	4.72 (1.85)*	5.05 (1.50)	0.385	−0.97 (−1.27 to −0.68)	<0.001
10 min after intervention	3.95 (1.93)*	5.23 (1.50)	0.002	−1.92 (−2.34 to −1.51)	<0.001
30 min after intervention	2.82 (2.01)*	5.41 (1.60)	<0.001	−3.23 (−3.74 to −2.72)	<0.001
Pain threshold at left SP9 (mean ± SD, *N*)					
Baseline	24.40 (8.01)	26.69 (7.39)	0.193		
30 min after intervention	21.67 (7.93)*	27.38 (8.18)	0.002	−3.41 (−5.02 to −1.81)	<0.001
Pain threshold at right SP9 (mean ± SD, *N*)					
Baseline	25.83 (7.98)	25.57 (6.98)	0.882		
30 min after intervention	21.33 (7.88)*	27.03 (8.07)*	0.002	−5.95 (−7.67 to −4.24)	<0.001
Temperature at CV4 (mean ± SD, °C)					
Baseline	34.37 (0.99)	34.11 (1.01)	0.255		
30 min after intervention	34.34 (1.15)	34.07 (0.90)	0.246	0.01 (−0.63 to 0.66)	0.970
Yes answer to ETCS1 (*n*[%])					
Baseline	35 (89.7)	36 (92.3)	0.692		
30 min after intervention	39 (100)	37 (94.9)	0.151	0.05 (−0.02 to 0.12)	
Yes answer to ETCS2 (*n*[%])					
Baseline	30 (76.9)	32 (82.1)	0.575		
30 min after intervention	39 (100)*	39 (100)*	1	0.00	
Yes answer to ETCS3 (*n*[%])					
Baseline	20 (51.3)	24 (61.5)	0.361		
30 min after intervention	33 (84.6)*	21 (53.8)	0.003	0.31 (0.11 to 0.50)	
Yes answer to ETCS4 (*n*[%])					
Baseline	22 (56.4)	22 (56.4)	1		
30 min after intervention	30 (76.9)*	21 (53.8)	0.003	0.23 (0.03 to 0.44)	

Small anomalies in subtraction are due to the effects of rounding. Negative between-group differences favour the AWA group.*: Compared with before intervention, *p* < 0.05. Analyses were performed on an intention-to-treat basis.AWA: acupressure wrist-ankle strap; NAWA: non-acupressure wrist-ankle strap; SD: standard deviation; SP9: Yinlingquan acupoint; CV4: Guanyuan acupoint. ETCS: Expectation and Treatment Credibility Scale; ETCS1: I am sure this treatment will relieve my pain; ETCS2: I think this treatment is reasonable; ETCS3: I will recommend this therapy to my friends; ETCS4: I believe this therapy can cure other diseases.

### 3.3 Secondary outcomes

Changes in the pain threshold at SP9 of both sides in AWA group differed significantly at each time point after intervention (Left: 95% CI, [−5.02 to −1.81], *p* < 0.001; Right: 95% CI, [−7.67 to −4.24], *p* < 0.001) (Table 3). In NAWA group, the change of pain threshold at left SP9 has no statistical significance (27.38 [8.18] vs 26.69 [7.39], *p* > 0.05) (Table 3), but has a significant increase of the pain threshold at right SP9 (27.03 [8.07] vs 25.57 [6.98], *p* < 0.05) (Table 3). There was no significant difference in the temperature at CV4 between the two groups after randomization (95% CI, [−0.63 to 0.66]; *p =* 0.970) (Table 3).

There was no statistically significant difference in the numbers of “Yes” answers to the 4 statements of ETCS questionnaire between the two groups before intervention (*p* > 0.05) (Table 3). After intervention, the numbers of “Yes” answers to ETCS3 and ETCS4 had significant differences between the two groups (ETCS3: 95% CI, [0.11 to 0.50]; ETCS4: 95% CI, [0.03 to 0.44]) (Table 3). As can be seen from Table 3, comparing with baseline, AWA group had statistically more “Yes” answers to ETCS2, ETCS3 and ETCS4 after intervention (*p* < 0.05), while NAWA group only had more “Yes” answers to ETCS2 (*p* < 0.05). These results demonstrate that AWA group had higher degree of satisfaction with the efficacy of the intervention (see Table 4).

**Table 4:** Onset time of analgesia reported by participants of the two groups.

Group	Number of participants who reported onset time	Fastest onset time reported (min)	Slowest onset time reported (min)	Average reported onset time (min)*
AWA (*n* = 39)	16	8	27	21.50 ± 3.65
NAWA (*n* = 39)	0	–	–	–

*Data are shown as mean ± standard deviation. Analyses were performed on an intention-to-treat basis.AWA, acupressure wrist-ankle strap; NAWA, non-acupressure wrist-ankle strap.Onset time of analgesia was defined as the time taken for the dysmenorrhea pain to be “obviously relieved” as reported by the participants.

### 3.4 Adverse events

During the intervention, there were no common adverse reactions such as hematoma and skin purple observed in both groups.

## 4 Discussion

The objective of this study was to assess the clinical efficacy of the AWA in alleviating primary dysmenorrhea through a single-blind RCT. We recorded the VAS scores, the onset time for “obvious relief” of dysmenorrhea pain, and changes in pain threshold at SP9 to evaluate the immediate analgesic effects of AWA on dysmenorrhea.

The findings demonstrated a significant reduction in VAS scores within the AWA group, with marked differences between the scores at 5, 10 and 30 min after intervention compared to baseline, which were statistically significant. The most substantial decline in VAS scores occurred from 10 to 30 min post-intervention. This reduction can be attributed to: (1) the lengthier measurement interval during this phase, enhancing the visibility of the strap’s efficacy; (2) the progressive increase in acupressure effects over the duration of the intervention. In contrast, the VAS scores in the NAWA group exhibited only a marginal decrease at 5 min post-intervention, lacking statistical significance, which suggests a possible placebo effect associated with NAWA.

Regarding the onset time for “obvious relief” of dysmenorrhea pain, 16 out of the 39 participants in the AWA group reported significant pain relief, with onset times ranging from 8 to 27 min and an average of 21.50 ± 3.65 min. The remaining 23 participants did not report “obvious relief,” possibly due to a more gradual alleviation of their pain. As indicated in Table 3, the VAS scores decreased from an initial 5.72 ± 1.75 to 2.82 ± 2.01 after 30 min of intervention, signifying that some participants continued to experience discomfort post-intervention.

The pressure pain threshold is a credible metric for validating the mechanisms of acupuncture, recognized as an effective and precise indicator for assessing pain (29).

In the AWA group, the pain threshold at SP9 on both sides exhibited a downward trend. Conversely, in the NAWA group, the pain threshold at the right SP9 displayed an upward trend, though no statistically significant changes were noted at the left SP9. Some studies have found that women with dysmenorrhea exhibit reduced sensitivity to subcutaneous and muscular electrical stimulation during menstruation (30–34), and the occurrence of dysmenorrhea appears to lower the pain threshold at skin tenderness sites (31, 32, 35, 36). Key reasons for the discrepancies between the results of this study and those of previously published studies (30–35) include: (1) women with dysmenorrhea exhibit heightened pain sensitivity compared to healthy individuals (31, 32, 34); (2) pain intensity during menstruation was measured in these studies without any intervention (30–35); (3) variations in the selection of experimental pain stimuli, such as the location and depth of application. These methodological differences complicate the drawing of linear conclusions due to the diverse range of painful stimuli, neglect of anatomical variations in pain areas, and the absence of dysmenorrhea as the “background” pain when testing pain thresholds outside the menstrual period. Some studies have suggested that dysmenorrhea may be associated only with somatic hyperalgesia of deep tissues, while central sensitization of skin pain is less evident (35). The pain experienced by individuals with dysmenorrhea is complex, subjective, and multidimensional, with significant interindividual variability in pain perception, further complicating the derivation of reliable conclusions.

We also monitored temperature changes at CV4 to explore the potential mechanisms of action of AWA. The results indicated no statistically significant differences in temperature at CV4, either pre- and post-intervention or between groups (*p* > 0.05). Previous research by Slater et al. (36) demonstrated that acupuncture at Sanyinjiao (SP6, corresponding to the Lower 2 compression point in this study) can enhance uterine artery blood flow, thereby alleviating dysmenorrhea pain. Other studies reported significant increases in skin temperature at CV4 following acupuncture at SP6 (37). In our study, the absence of temperature changes at CV4 might be attributed to the limited duration of acupoint pressure (30 min) and the timing of temperature measurements post-intervention. Nonetheless, these findings suggest that the immediate analgesic effects of AWA do not derive from increased temperature at CV4 through improved uterine artery blood flow as induced by needling stimulation at SP6.

In this trial, the ETCS questionnaire was employed to assess patients’ expectations and satisfaction with the intervention’s efficacy. The results indicated that the AWA group reported a significantly higher degree of satisfaction with the intervention’s efficacy compared to the NAWA group, suggesting that AWA was substantially more effective than the sham control.

AWA represents a physical therapy approach that is effective, non-toxic, and devoid of side effects. It is simple to administer and can be used repeatedly. Further research should explore the effectiveness of AWA for other conditions.

## 5 Limitations

This study is subject to several limitations. Firstly, the investigation was confined to observing the effects of a single 30-min treatment session, specifically focusing on three time points during the intervention. The primary aim was to assess the immediate analgesic effects. Future research is necessary to explore the long-term efficacy of this device and to ascertain its safety and acceptability for frequent use over extended periods. Secondly, the recruitment of participants was limited to students from a university with a high proportion of female students in order to secure a sufficient sample size within a specific timeframe and to ensure participant compliance. This selection criterion may affect the external validity of the findings. Future studies should aim to include a more diverse participant pool to verify the efficacy of the AWA more broadly. Thirdly, the potential impact of the treatment may have been overestimated due to the lack of follow-up assessments. Fourthly, the VAS of the control group exhibited a decrease at the 5th minute which could be attributed to a placebo effect. To further investigate this phenomenon, it is recommended to increase the number of blank controls and trials in subsequent experiments.

## 6 Conclusion

This trial substantiated our hypothesis that the AWA provides immediate analgesic effects for patients suffering from primary dysmenorrhea. The AWA represents a safe and efficacious intervention for alleviating abdominal pain associated with this condition. As a non-invasive physical therapy, it offers a self-administered, non-pharmacological option for pain relief in patients with primary dysmenorrhea. The clinical relevance of AWA could be further enhanced by extensive research to validate its effectiveness for additional medical conditions.

## Data availability statement

The original contributions presented in the study are included in the article/supplementary material, further inquiries can be directed to the corresponding authors.

## Ethics statement

The studies involving humans were approved by China Ethics Committee of Registering Clinical Trials (ethics reference: ChiECRCT20190037). The studies were conducted in accordance with the local legislation and institutional requirements. The participants provided their written informed consent to participate in this study. Written informed consent was obtained from the individual(s) for the publication of any potentially identifiable images or data included in this article.

## Author contributions

SZ: Data curation, Supervision, Writing – original draft, Writing – review & editing. CW: Data curation, Software, Supervision, Writing – review & editing. YR: Data curation, Formal analysis, Methodology, Supervision, Writing – review & editing. YL: Writing – review & editing, Conceptualization, Resources, Validation. RM: Resources, Data curation, Writing – review & editing. FF: Funding acquisition, Resources, Validation, Writing – review & editing. QZ: Methodology, Supervision, Writing – review & editing.
